# Microstructure and Mechanical Properties of MIG Welds between 6252 Armor Steel and Q550D HSLA Steel

**DOI:** 10.3390/ma15249043

**Published:** 2022-12-17

**Authors:** Xiangyu Xu, Gang Wang, Runbo Zhang, Guangjun Zhang

**Affiliations:** State Key Laboratory of Advanced Welding and Joining, Harbin Institute of Technology, Harbin 150001, China

**Keywords:** 6252 armor steel, Q550D HSLA steel, mechanical property, microstructure

## Abstract

The microstructure and mechanical properties of the welded joints of 6252 armor steel and Q550D high-strength low-alloy (HSLA) steel welded by MIG welding were studied. ER70S-G and ER140S-G were used as fillers to obtain welded joints with good formation and no faults. The joint microstructure (OM) analysis showed that a large Widmanstätten structure was observed at the fusion line on the Q550D side, and the apparent grain sizes changed on the 6252 side. Cylindrical ferrite growth along the bainite grain boundary was observed in the ER70S-G filler weld zone, while the ER140S-G filler weld zone was occupied by lower bainite structures. The XRD phase analysis showed that more Fe-Ni-Cr compounds and less ferrite were formed in the ER140S-G filler weld. The hardness test showed that the hardness of the HAZ on the 6252 side was significantly higher than that of the BM and the WZ, and the welded joint obtained by the ER140S-G filler had a higher hardness. The tensile strength test showed that WZ (>772 MPa) had a higher strength than Q550D BM, and the tensile fracture (SEM) was primarily a ductile fracture. The impact test results showed that the welded joint had better impact resistance at room temperature, but the impact absorption energy of the weld and the heat-affected zone was strongly affected by changes in temperature, and brittle fracture occurred easily at low temperatures.

## 1. Introduction

With the rapid growth of submarines, aerospace and military protection, the demand for ultra-high-strength steel is on the rise. As a result of the continuous improvement of the manufacturing level of the equipment, traditional high-strength steel is no longer able to meet the protection and load-bearing requirements of certain structures and is gradually being replaced by new steel. For instance, 6252 is a newly developed 7-level armored steel [1,2]. Complex hot-rolling and tempering processes produce an extremely fine bainite matrix, and the Ni-Cr precipitation reinforcement phase provides unmatched high strength, high rigidity and high hardness [3,4]. However, because of its extremely high hardness and fine grains, excessive grain growth will inevitably occur due to the thermal welding cycle and the filling material. Coarse grain joints tend to have more welding defects, such as cracking and excessive deformation of the weld, resulting in a decrease in protective performance [5,6,7,8].

Q550D high-strength low-alloy (HSLA) steel is smelted by adding an alloy solid solution on the basis of carbon structural steel [9]. By reinforcing the solution, the inner ferrite of Q550D is deformed, the hardenability and tempering stability of the steel are improved, and the strength of the steel is enhanced [10]. Compared to 6252 armor steel, Q550D has a lower sensitivity to weld cracks and better weldability, thus it is widely used in the manufacture of hydropower plants, engineering machinery, vehicles and buildings with high welding requirements [11,12].

In the area of protective equipment fabrication, high-quality, expensive protective materials are generally only used close to the hazardous points of the supporting structures for economic reasons and often involve dissimilar connections. MIG welding is widely used in production lines for automatic welding [13,14]. Compared to MAG welding, which is commonly used for welding high-strength steel, MIG welding uses pure argon protection to reduce welding heat input, making it easier to control grain growth, welding deformation, and joint properties. However, when welding different high-strength steels, the different thermal properties of the materials can easily cause defects like deformation and softening, including some subsequent effects, such as cracks causing a sudden drop in weld strength, softening decreasing the hardness value in the WZ, and the thermal cycle increasing the low temperature ductile–brittle transition temperature of the material. These conditions lead to poor mechanical properties of the joints and the deterioration of the tissue composition. As such, its microstructure and mechanical properties must be evaluated [15,16,17].

Zhu et al. [18] analyzed the welding performance of Q550D high-strength steel by testing the maximum hardness and Y-type cracking in the HAZ. The results showed that the maximum hardness (HV10) of the HAZ of the Q550D steel plate was 297 HV, and there was no cracking of the weld surface and no cracking of the cross-section. Shen et al. [19] studied the buckling behavior of the welded thin wall rectangular hollow section Q550D under compression bending load and obtained the ultimate bearing stress of the welded joints. Wang et al. [20] studied the development of the microstructure of 6252 steel under high-speed impact. By designing the impact velocity respectively, the phase transition evolution of the 6252 armor steel was martensite → austenite → bainite + M/A island + martensite, whereas research into the weldability of these materials, including the connection of dissimilar materials of 6252 armor steel, is new.

However, other studies on the weldability of high-strength steel similar to 6252 armor steel have found that the mechanical properties of the joints, especially in the connection of dissimilar materials, are significantly correlated with changes in the performance and microstructure of the materials on the high strength and high hardness side. In addition, armored steel materials are more likely to exhibit a brittle fracture in low-temperature environments after the welding heat cycle. Dong et al. [21] studied the joint performance of HSLA steel under various thermal weld inputs using tungsten inert gas welding (TIG). By limiting the formation of martensite, the welding heat input of HSLA steel into the gas tungsten arc welding process was reduced, and the hardness of the heat-affected zone (HAZ) was improved, which reinforces the resistance of the HAZ. At the same time, it was concluded that the reduction in impact toughness in the HAZ was caused by grain enlargement and the unequal distribution of carbides and inclusions.

Mustafa Tümer et al. [22] studied the microstructures and mechanical properties of welds consisting of 20 mm thick S1100MC Ultra-High-Strength Steel (UHSS) plates (>1200 MPa) thermally laminated and directly quenched. A large amount of martensite was observed in the CGHAZ by using low-matching solid wire and the MAG welding method. The results of the hardness tests showed that the hardness of the FGHAZ exceeded that of the fusion zone, and the impact toughness of the weld zone was greatly enhanced relative to the base metal [23,24,25,26,27].

So far, few studies have been carried out on steels with a tensile strength of more than 1700 MPa, and most previous weldability studies have concentrated on a similar welding field. It is of great importance to investigate the microstructure and mechanical properties of dissimilar UHSS welded joints to save production costs and improve the level of military protection. However, the microstructure and properties of the dissimilar welded joints of 6252 are still uncertain. In order to provide some references for the safety of 6252 armor steel dissimilar MIG welded joints and the welding method in practice, this paper uses MIG welding 6252 armor steel and Q550 dissimilar butt joints to study the welding quality, microstructure, hardness, tensile strength and low-temperature ductile–brittle transition of 6252 dissimilar joints filled with two kinds of welding wires under this method.

## 2. Experimental Procedures

The materials studied are 6.3 mm thick 6252 armor steel and Q550D low-alloy structural steel. The fillers are the welding wires ER70S-G and ER140S-G. Table 1 illustrates the composition of the base metals and the filling materials. According to the AWS specifications, a conventional V-groove was cut from a 250 mm × 100 mm plate and cleaned, ground and affixed. The mechanical characteristics of the welding materials are given in Table 2.

**Figure 1:**
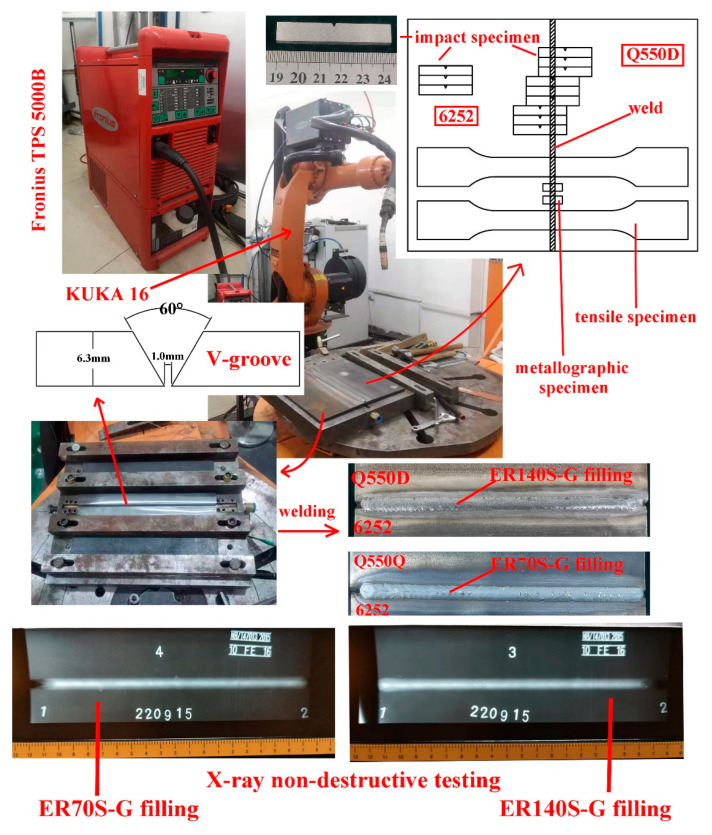
Flow chart of the experimental process.

The MIG dissimilar joint test of the 6252 armor steel and Q550D low-alloy steel was carried out. The welding energy supply was Fronius TPS5000 B, and a KUKA KR16 robot was used to carry out the automatic welding. The weld formation is shown in Figure 1. The welding parameters are shown in Table 3. To ensure the quality of subsequent test pieces, a small XXG-2505 X-ray flaw detector was used to inspect the weld, and an LK-LED33T film viewer was used to analyze the location of weld defects. No obvious weld defects, such as pores and cracks, were identified in the MIG welded joints, and the defect detection rating was the first quality, which met the requirements for the production of tensile, impact and metallographic samples.

## 3. Results and Discussion

### 3.1. Microstructure

**Figure 2:**
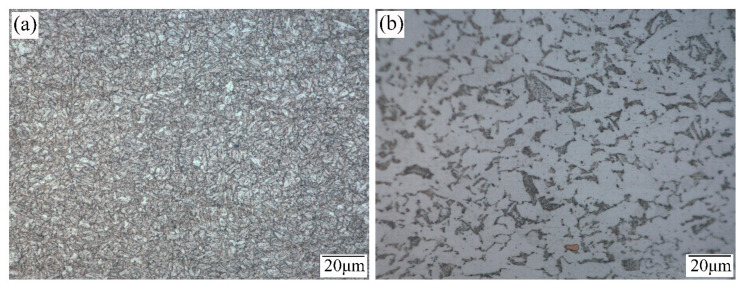
Microstructure of 6252 armor steel and Q550D at 1000×. (**a**) 6252 (**b**) Q550.

The microstructure of the base metal of the 6252 armor steel is shown in Figure 2a, which is a granular bainite structure after tempering and cooling in air. The bainite grain size is very fine, corresponding to the nominal 5 µm ASTM mean linear intercept grain size, which is also the reason for its ultra-high strength. Figure 2b shows coarse-grained bainite and polygonal ferrite precipitation along the grain boundary in the Q550D microstructure. To investigate the effect of dissimilar metal hardening on the microstructure and mechanical properties of welded joints, the microstructure and fracture morphology of the weld were observed using an optical microscope and a scanning electron microscope, and the tensile strength, low-temperature impact toughness and Vickers microhardness of the weld were tested.

**Figure 3:**
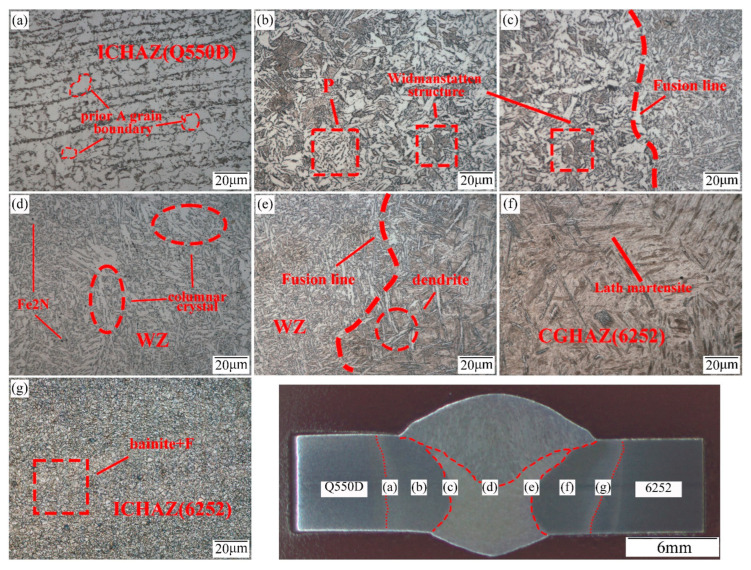
Microstructure of the dissimilar welded joint (ER70S-G): (**a**) intercritical HAZ (Q550D), (**b**) coarse-grained HAZ (Q550D), (**c**) fusion zone (Q550D), (**d**) weld zone, (**e**) fusion zone (6252), (**f**) coarse-grained HAZ (6252), and (**g**) intercritical HAZ (6252).

Figure 3 shows the optical microstructure of the joint of the 6252 armor steel and Q550D steel plate welded by ER70S-G. The microstructure of the weld is granular bainite, with some nonmetallic inclusions, and a great ferrite zone can be observed (zone d). The remainder of the ferrite was distributed along the columnar crystal in the shape of a needle, and the columnar crystal can be clearly seen in the micro-metallography photos. The fusion zone on the left side of the weld (Q550D) is shown in Figure 3c. Because it is close to the weld and has a high heat input, massive proeutectoid ferrite precipitates developed along the grain limit, and austenitic grains grew significantly [3,5,20]. After cooling, a Widmanstätten structure was produced at the fusion line of Q550D, and the matrix structure is pearlite (zone b). With increasing distance from the center of the weld, the HAZ particle size on the left side of the weld (Q550D) was progressively refined, and the maximum temperature of the heat cycle was gradually lowered [5,13]. As shown in Figure 3a, the fine-grained HAZ (FGHAZ) was far away from the weld center. Compared with the coarse-grained area, the grain size of the bainitic structure was reduced, and less ferrite precipitated between the grains, and as a result, the previous austenitic grain boundary can be clearly observed [23].

The microstructure on the right side of the weld (6252) is shown in Figure 3e–g and the distribution of the HAZ on the right side is similar to the one on the left, with a coarse-grained HAZ (CGHAZ) near the weld and a fine-grained HAZ far from the center of the weld. The zone of the fusion line (zone e) is mainly composed of dendrites, which grew vertically on both sides of the fusion line [10]. The peak temperature of the CGHAZ thermal cycle was greater than 1100, releasing a great deal of structural stress, and a large amount of lath martensite and a small amount of bainite were formed after cooling, which may be due to its strong thermal conductivity. Likewise, as the distance from the center of the weld increased, the peak temperature of the thermal cycle decreased, and the grain size of the HAZ gradually refined (zone f). The martensite structure was gradually replaced with pearlite, and the ferrite precipitation decreased [13]. The microstructure of this fine-grained area (zone g) consists of granular bainite and a small amount of ferrite, with a grain size equivalent to that of the base metal.

**Figure 4:**
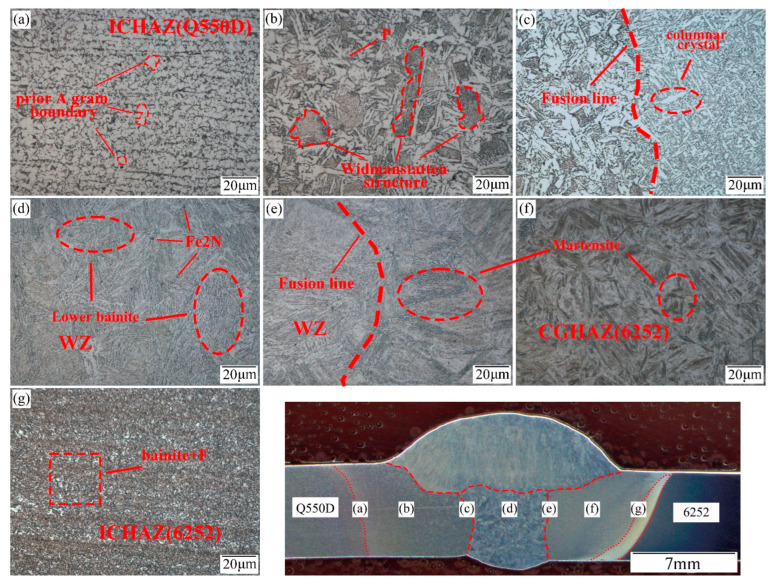
Microstructure of the dissimilar welded joint (ER140S-G): (**a**) intercritical HAZ (Q550D), (**b**) coarse-grained HAZ (Q550D), (**c**) fusion zone (Q550D), (**d**) weld zone, (**e**) fusion zone (6252), (**f**) coarse-grained HAZ (6252), and (**g**) intercritical zone (6252).

Figure 4 shows the optical microstructure of the 6252 armor steel and Q550D steel sheet welded by ER140S-G. In contrast to the lower strength wire, the welded joint using the higher strength wire produced a large quantity of lower bainite structures (zone d) at the weld, and an aggregation distribution of ferrite was not observed. In addition, the lath martensite near the side fusion line of the 6252 armor steel filled with ER140S-G wire had coarser grains and fewer M-A constituents [14,28]. For the HAZ on both sides, the distribution of the seam microstructure obtained using different welding wires was fundamentally coherent.

**Figure 5:**
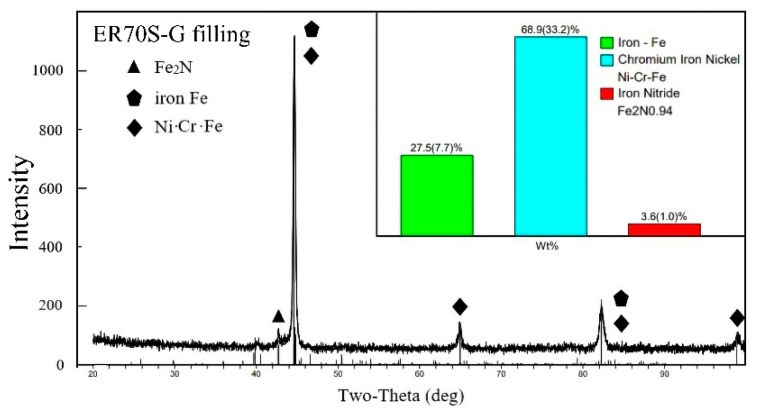
XRD phase detection spectrum (ER70S-G filling).

**Figure 6:**
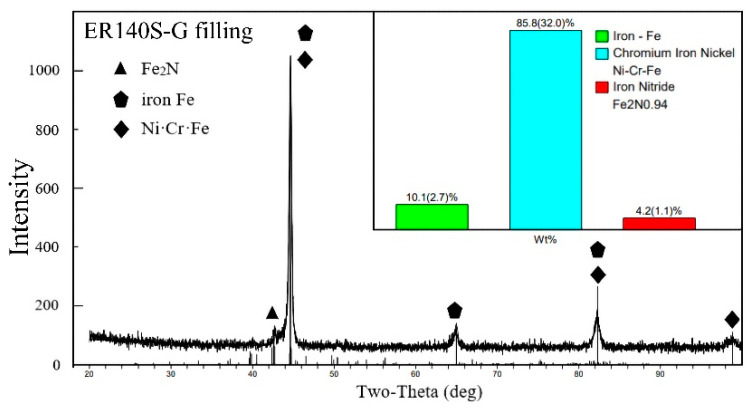
XRD phase detection spectrum (ER140S-G filling).

The XRD phase analysis was carried out on the weld zone, and the spectrum of the phase results is shown in Figure 5 and Figure 6. The interaction between Cr, Ni and Fe in the melt was intense, and a large number of Ni-Cr-Fe intermetal compounds formed in the weld area. In addition, a low level of Fe_2_N (ξ phase) was also generated in the weld. It can be seen in Figure 3d and Figure 4d that Fe_2_N was mainly dispersed in the weld area as second-stage particles, while various intermetallic compounds were generated within the bainite grains of the weld, which are not easily observed on metallographic photos. Additionally, more intermetal compounds (85.8%), such as Ni-Cr-Fe, were formed in the ER140S-G filler weld, and the level of ferrite in the weld decreased (10.1%), which corresponds to the metallographic observation.

### 3.2. Hardness

**Figure 7:**
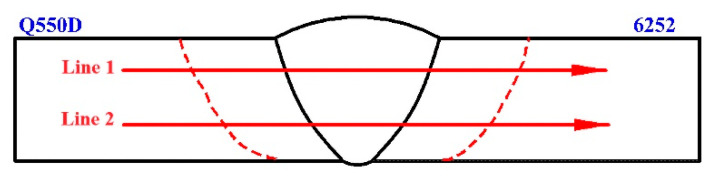
The measured hardness location.

**Figure 8:**
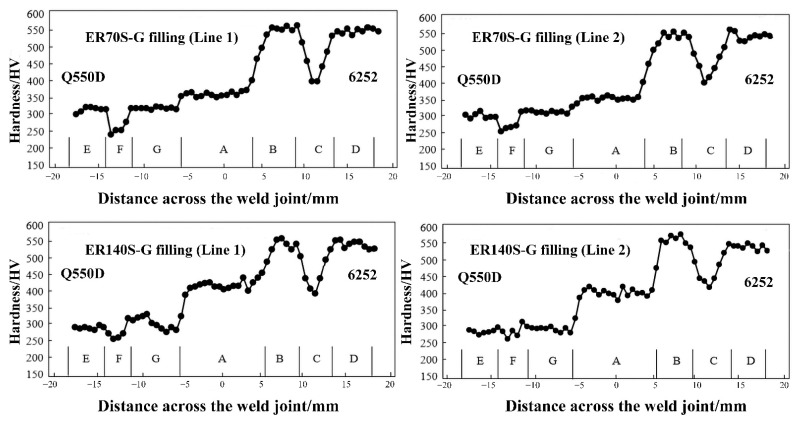
Hardness distribution of the weld section.

The Vickers hardness measurement locations are depicted in Figure 7. Figure 8 shows the hardness distribution of the 6.3 mm thick dissimilar welded joint. The thermal cycle of the welding process alters the microstructure, causing a modification of the interfacial hardness [17,20].

When using ER140S-G as a welding wire, lower bainite formed within the weld area, which made the weld zone hardness (zone A) much greater than the base metal zone Q550D (zone E), but still below that of the 6252 armor steel (zone D), and its Vickers hardness was 380–450 HV, which represents approximately 70% of the hardness of the 6252 armor steel. However, the metal deposited from the ER70S-G welding wire had a lower resistance, and the Vickers hardness was 340~360 HV. With the exception of the weld zone, the hardness of the fusion zone on the 6252 side (zone B) was slightly higher than that of the base metal (zone D). Because of the high peak temperature of the thermal cycle, the speed of rapid cooling, as well as the influence of the tempering elements Mn and Mo, which produce a large amount of martensite slat, the maximum hardness can be up to 570 HV. Zone C is the HAZ zone, where part of the bainite reverted to austenite and ferrite precipitates at the border under the influence of a lower thermal cycle temperature, and the size of the grain in the area was slightly larger than the base metal, while the hardness was significantly lower than that of the melting zone (zone B) and the base metal (zone D) on either side.

Different from the 6252 armor steel side, the hardness value on the Q550D side had virtually no change, except that the hardness value at the edge of the HAZ (zone F) decreased slightly, and the hardness of the melting zone (zone G) was equivalent to that of the basic metal (zone E). Grain growth may lead to the softening of the fusion zone and counterbalances the hardening effect, showing that the hardness level is comparable with that of the base metal (zone E).

### 3.3. Tensile Strength

**Figure 9:**
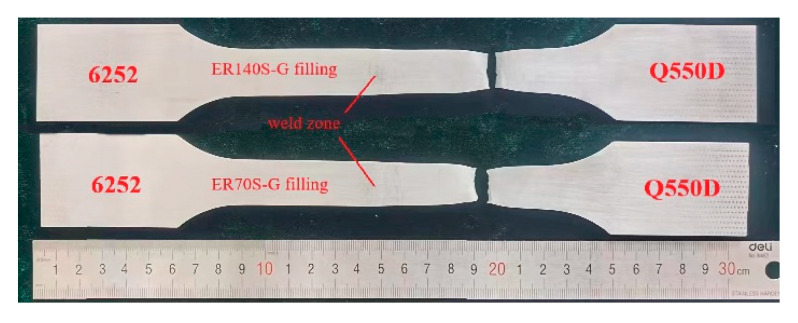
Failure zones of dissimilar welds of 6252/Q550D.

Table 4 illustrates the tensile properties of the 6252/Q550D armor steel welds. The fracture position of the welded specimens with both types of welding wire was within the zone of the low-alloy base metal Q550D, away from the weld, and the extension after fracture was basically identical to that of the base metal Q550D. The location where the tensile test piece failed is shown in Figure 9. Due to the large resistance difference between 6252 armor steel and the low-alloy Q550D steel, the metal deposited in the weld area was doped with reinforcement elements of 6252 and welding wire so that the weld resistance could easily exceed that of the alloy steel Q550D. Although the strength of the CAZ tends to decrease due to grain growth during the thermal welding cycle, the carbon content of the Q550D is relatively low, and dispersal particles generated by a variety of alloy elements enhance the HAZ. The strength of the welded joint is greater than that of Q550D low-alloy steel and meets the strength requirements. Furthermore, by observing and measuring the size of the weld position following a tensile fracture, there was no apparent tendency for plastic deformation at the weld, indicating that the yield strength of the weld was higher than 772 MPa.

**Figure 10:**
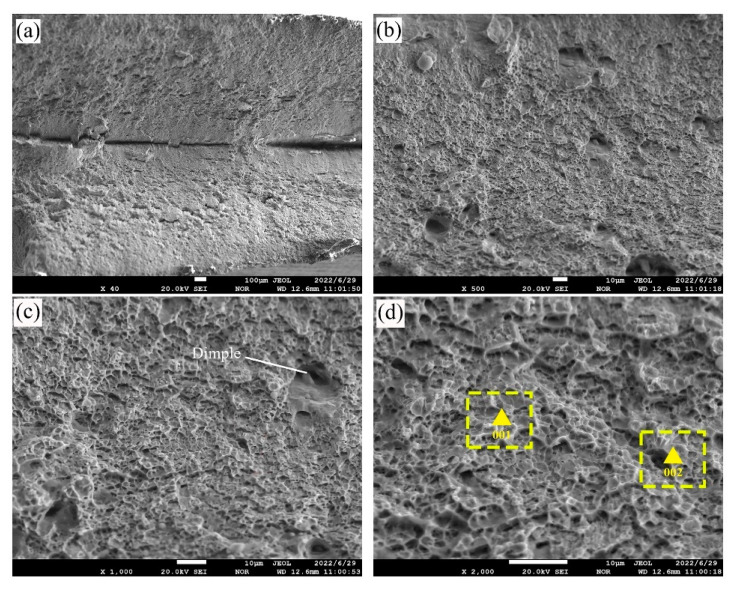
Fractographs of similar welds of 6252 Armor steel. (**a**) 40× (**b**) 500× (**c**) 1000× (**d**) 2000×.

Figure 10 shows the morphological characteristics of the tensile test piece failure (filled with ER140S-G) under SEM. A peak fracture can be clearly observed on the macro image (40), indicating that the primary failure mode was a ductile fracture and that there is no obvious segregation zone [2,7,17], indicating that the location of the fracture was less affected by the welding heat cycle and that the location of the failure was away from the HAZ. Larger dimples are visible in Figure 10c, and the results of the tensile properties are consistent with the observed failure. Next, the element content of the tensile fracture surface was tested, and the test results are shown in Table 5, which correspond to the structure of steel Q550D [18,19]. Consequently, it was determined that the fracture location of the tensile specimen was the base metal Q550D, away from the weld, which demonstrated that the resistance of the heat-affected zone on the Q550D side was also superior to that of the base metal on that side.

### 3.4. Impact Toughness

**Figure 11:**
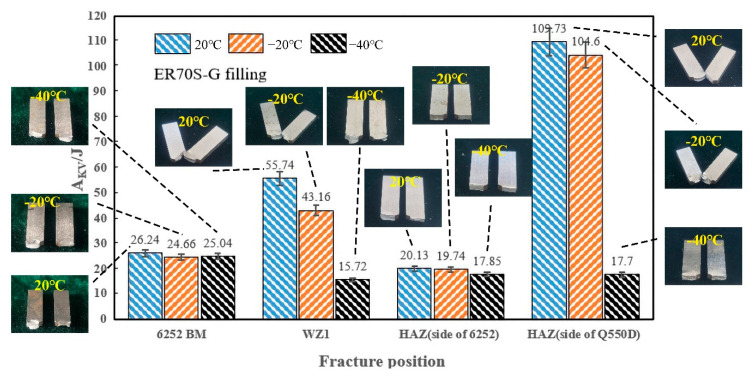
A_KV_ of various specimens at different temperatures (ER70S-G).

**Figure 12:**
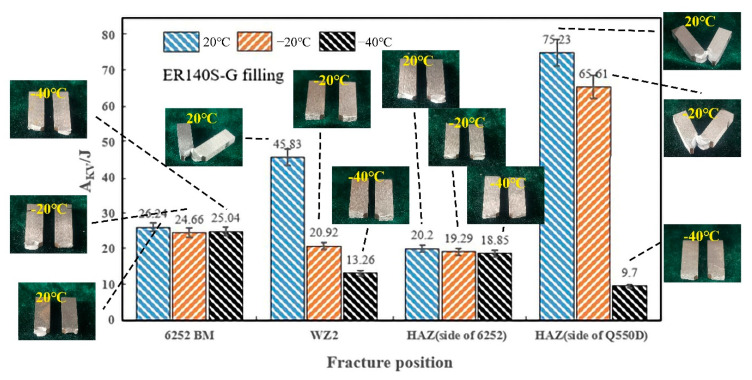
A_KV_ of various specimens at different temperatures (ER140S-G).

The value of the impact absorbing energy at different temperatures at each position of the welded joint filled with two types of welding wires is shown in Figure 11 and Figure 12. The impact toughness of the 6252 (6252 BM) armor steel base material at −40 °C may still be maintained at the same level (25 J) as in normal temperatures, and the toughness was improved compared with 6252; thus the impact absorbing energy of the weld at room temperature was improved (56 J). The weld zone (WZ) had a variety of alloying elements and was not strengthened after welding. As the impact test temperature decreased, the intermolecular force decreased, and the elasticity decreased, which indicates that the impact absorption energy decreased (16 J) with decreasing temperature. This is particularly apparent on the HAZ side of the Q550D low-alloy steel. Although the grains of the HAZ on the 6252 side grew, a large amount of martensite slat was generated near the fusion line. The impact absorption energy decreased significantly, thus reducing impact toughness, which is compatible with the results of the hardness distribution of the welded joint in Figure 8.

By comparing the two types of welded joints, the energy absorbed by the impact of the welded joint using welding wire ER140S-G was smaller after cooling (46 J), which could be due to the lower elongation of this welding wire, causing the toughness to diminish after cooling. The fragile ductile transition temperatures on the 6252 (BM, HAZ) side of the two welded joints were less than −40 °C, and the ductile–brittle transition temperatures of the HAZ on the Q550D side were in the range of −20 to −40 °C. The fragile ductile transition temperature of the ER70S-G (WZ1) filled weld area was between −20 and −40 °C, whereas the brittle transition temperature of the weld area ER140S-G (WZ2) occurred before the temperature dropped to −20 °C.

Figure 11 shows the failure mode of the 6252 armor steel base metal and ER70S-G filled impact samples. The impact specimen of the 6252 base metal (6252 MB) was completely broken at all temperatures, and the failure mode was a ductile rupture. The instantaneous fracture area was small, with the shear lips on both sides clearly visible [16,23]. The weld area specimen (WZ1) cracked along the weld and surface of the Q550D seam at room temperature and −20 °C and was not completely shattered due to increased plasticity. So, the fracture pattern was ductile, and the fracture was tearing. When the temperature dropped to −40 °C, the specimen was completely broken, and the specimen fractured along the center of the weld, but the fracture shape was still predominantly ductile. The specimen with the 6252 side HAZ showed an obvious brittle fracture at all temperatures. The fracture surface was occupied by the instantaneous fracture area, and only a small dilation area could be observed at ambient temperature. This is because the martensite lath in the soldering zone significantly reduced the low-temperature toughness of the material and increased the ductile–brittle transition temperature by 6252 armor steel [17]. The samples from the HAZ on the Q550D side were not broken prior to −20 °C, showing an evident ductile fracture. However, when the temperature was reduced to −40 °C, the toughness of the test pieces decreased rapidly, and the fracture of the test pieces exhibited a brittle fracture.

Figure 12 indicates the failure mode of the impact samples filled by ER140-G. The Q550D HAZ specimens had a similar performance at different temperatures, which could be caused by the high residual stresses produced during the cold rolling of the low-alloy steel Q550D. In addition, the interaction between interstitial atoms and grain dislocations and boundaries was reinforced at low temperatures, which interfered with the dislocation motion and weakened the deformation adaptability, resulting in low toughness at low temperatures [16,29]. However, 6252 armor steel is primarily manufactured by hot rolling, and the residual stress level is low. Consequently, the impact toughness of the HAZ on the 6252 side did not change significantly with temperature. The toughness transition of the HAZ is not affected by the type of filling wire but depends on the microstructure and properties of the heated base metal. The welded specimen (WZ2) showed a brittle break at −20 °C, and the ductile–brittle transition temperature was higher than the ER70S-G (WZ1) weld specimen, which corresponds to the results for the absorbed impact energy (Figure 11 and Figure 12) under various temperatures.

Additionally, changing the grain size affects toughness and failure modes at different joint positions, and the effect of this on the materials on either side is different. In general, the finer the grain structure, the greater the strength and strain resistance of the material, and vice versa [10,20]. This rule is particularly obvious in the HAZ and WZ on the 6252 side. Even if the high toughness from the lath martensite phase is formed on the 6252 side of the fusion line, grain growth also makes the impact toughness of the area inferior to that of the 6252 BM. In the HAZ on the Q550D side, grains also grow, but the resulting network structure causes the region to achieve higher toughness at ambient temperature and is highly brittle when the temperature drops.

## 4. Conclusions

In this paper, the welding of the 6252 armor steel and low-alloy steel Q550D was performed by MIG welding, and the microstructure and mechanical properties of the welded joints were analyzed for two types of welding wires with different strengths used as filling materials. The tensile test showed that the strength of the weld conformed to the operating requirements, and further study on the resistance of UHSS welded joints is necessary for the future. The results indicated that the hardness, low-temperature toughness and microstructure of the weld are affected by the strength of the welding wire. The specific conclusions are set out below:

(1) Whether ER70S-G or ER140S-G was filled, dissimilar welded joints of 6252 armor steel and Q550D low-alloy steel with good formation and no defects could be obtained by the MIG welding process, but there were some differences in the microstructure of the weld area, while the microstructure of other areas of the joint was similar. The weld strength was much higher than that of the Q550D low-alloy steel base metal, and the two welding wires could ensure that the weld’s yield strength exceeded 772 MPa.
(2) The welded joint filled with ER140S-G had a higher hardness of approximately 380~450 HV, whereas the hardness of the HAZ was not affected by the type of welding wire. Additionally, the HAZ on the 6252 armor steel side was hardened significantly, up to 570 HV.
(3) Compared to ER140S-G, ER70S-G filler joints had superior impact toughness. The type of welding wire had little effect on the impact toughness at low temperatures on the 6252 armor side but had a significant impact on the ductile–brittle transition temperature of the weld area and low-alloy Q550D side.

## Figures and Tables

**Table 1:** Chemical composition.

Material	Chemical Composition/Wt. %								
	C	Si	Mn	Ni	Mo	Ti	Al	Cr	Fe
6252	0.28	0.16	1.40	0.016	0.28	0.062	0.051	0.030	Balance
Q550D	0.18	0.52	1.56	1.80	0.060	0.052	0.032	1.50	Balance
ER70S-G	0.07	0.67	1.49	0.015	0.001	0.17	—	0.029	Balance
ER140S-G	0.11	0.60	1.80	2.63	0.80	—	—	0.054	Balance

**Table 2:** Mechanical properties of the welding materials.

Materials	0.2% Yield Strength/MPa	Ultimate Tensile Strength/MPa	A_kv_ (20 °C, 4.5 mm)/J	Elongation/%	Hardness Value/HV
6252	1509.33	1786.66	26.24	3.10	523.0
Q550D	552.35	784.34	46.74	20.5	290.4
ER70S-G	460.82	565.71	90.13	30.5	—
ER140S-G	1020.63	1135.79	69.54	16.67	—

**Table 3:** Welding parameters.

Welding Sequence	Joint Design	Current/A	Wire Feeding Speed/(m·min^−1^)	Voltage/V	Welding Speed/ (m·min^−1^)
1	Single V groove	147	0.3	23	0.35
2		163	0.5	27	0.4

**Table 4:** Tensile properties of different dissimilar steel welds.

Specimen	Tensile Properties			Failure Location
	0.2%YS/MPa	UTS/MPa	%EL	
6252/Q550D (ER70S-G)	551 ± 5	767 ± 7	21	Base metal (Q550D)
6252/Q550D (ER140S-G)	556 ± 4	772 ± 7	21	Base metal (Q550D)

**Table 5:** Elemental composition.

Element	Spectrum 001 (At%)	Spectrum 002 (At%)
Fe	94.39	94.34
Mn	1.54	1.64
Al	0.01	0.03
Si	0.51	0.47
Ni	1.92	1.87
Cr	1.63	1.65
